# Restoration of Sagittal Alignment and Pulmonary Function With Percutaneous Vertebral Body Augmentation for Painful Osteoporotic Vertebral Compression Fractures: A Systematic Review

**DOI:** 10.7759/cureus.83694

**Published:** 2025-05-07

**Authors:** Hanne H Jørgensen, Mikkel Ø Andersen, Tove F Frandsen, Line A Wickstrøm, Benjamin Kostic, Leah Y Carreon

**Affiliations:** 1 Spine Centre of Southern Denmark, Lillebaelt Hospital, Kolding, DNK; 2 Department of Design and Communication, University of Southern Denmark, Kolding, DNK; 3 College of Arts and Sciences, University of Louisville, Louisville, USA; 4 Department of Regional Health Research, University of Southern Denmark, Odense, DNK

**Keywords:** osteoporotic fracture, postural balance, pulmonary function, sagittal alignment, vertebral augmentation, vertebral compression fracture

## Abstract

Sagittal spinal malalignment is associated with functional disability. Therefore, a key question in treating patients with painful osteoporotic vertebral compression fractures (OVCFs) is whether vertebral augmentation improves sagittal alignment and its associated outcomes. In a systematic literature review based on interventional and observational studies, we evaluated the effect of percutaneous vertebral body augmentation on sagittal alignment and pulmonary function in patients with painful OVCFs. In November 2022 and December 2023, we searched for relevant studies in Medline, Embase, SCOPUS, Web of Science, the Cochrane Central Registry of Controlled Trials, and five trial registries. In total, 15 cohort studies and two non-randomized clinical trials met our inclusion criteria. Participants in these studies had painful OVCFs treated with vertebral body augmentation. Sagittal alignment outcomes from nine articles represented 456 participants with mean ages from 69.3 to 80.8 years, and pulmonary function outcomes from eight articles represented 343 participants with mean ages from 69.1 to 75.7 years. The quality assessment tool for quantitative studies by the Effective Public Health Practice Project assessed the risk of bias (RoB). Mean pre- and postoperative outcome values were calculated for all included studies and those without a high RoB. None of the sagittal alignment parameters improved after vertebral augmentation, and no additional deformity was detected. Vertebral augmentation positively affected pulmonary function, as measured by the percentage of predicted values, and reduced perceived pain levels and functional disability. Data in the included studies were incomplete due to variability in chosen outcomes and follow-up time points. The incomplete data curtailed our data analysis and only allowed cautious conclusions. Variations in study populations and protocols highlight the need for standardized reporting and follow-up in future research.

## Introduction and background

Appropriate management of painful osteoporotic vertebral compression fractures (OVCFs) remains an ongoing topic of debate. Worldwide, the estimated number of individuals with a 10-year probability of a major osteoporotic fracture is expected to increase from 158 million in 2010 to 371 million in 2040 [1], with vertebral fractures accounting for 16% of fragility fractures in Europe [2].

Effective pain management is a crucial step toward restoring normal activities of daily living in patients with painful OVCFs. Consequently, randomized controlled trials (RCTs) [3-5] and a Cochrane review [6] focused on pain reduction and safety of vertebral body augmentation for the treatment of OVCFs.

Spinal malalignment due to OVCFs is associated with functional disability [7,8] as the spine becomes malaligned due to a combination of aging [9], osteoporosis, and OVCFs [10,11]. OVCFs, in turn, are associated with decreased quality of life [12,13], smaller chest volume, and decreased pulmonary function [14-16].

Sagittal spine alignment is strongly associated with clinical outcomes [17]. Radiographic parameters were measured in only three of the nine most cited RCTs on vertebral body augmentation, and none of the studies included outcomes on sagittal alignment [18]. In addition, a recent systematic review on the impact of vertebral augmentation on pulmonary function is not available. We, therefore, conducted a systematic literature review on radiographic outcomes from both interventional and observational studies to determine if percutaneous vertebral body augmentation (1) protects or restores sagittal alignment and (2) improves pulmonary function in patients with painful OVCFs.

## Review

Methodology

Study Design and Research Question

The protocol for this systematic review was registered on September 9, 2022, on the International Prospective Register of Systematic Reviews (PROSPERO) and https://www.crd.york.ac.uk/PROSPERO/view/CRD42022349164 is the location it can be accessed at. Prior to registration, a search on PROSPERO was performed to confirm that no identical literature reviews were in progress. The critical appraisal tool, a Measurement Tool to Assess Systematic Reviews (AMSTAR 2) [19], was used as a guide to conduct the review, and it is presented according to the Preferred Reporting Items for Systematic Reviews and Meta-Analyses (PRISMA) guideline [20].

The review was designed to estimate any effect of vertebral body augmentation on sagittal alignment and pulmonary function using the PICO model: (P) populations of individuals with painful OVCFs; (I) interventions in which bone cement is injected into the vertebrae percutaneously; (C) comparison to individuals receiving non-surgical treatment (alternatively, two vertebral augmentation procedures are compared, or a longitudinal cohort study includes only before and after intervention comparison); and (O) outcome measures associated with spinal sagittal alignment and pulmonary function.

Information Sources and Searches

We searched five bibliographic databases for published and accepted articles: Medline, Embase, SCOPUS, Web of Science, and the Cochrane Central Registry of Controlled Trials. In addition, we searched five trial registries for relevant ongoing studies and clinical study reports: ClinicalTrials.gov, the WHO International Clinical Trials Registry Platform, the EU Clinical Trials Register, the International Standard Randomised Controlled Trial Number registry, and the Medical Research Council Clinical Trials Unit.

T.F.F., an experienced information specialist, reviewed the multi-stranded search strategy before H.J. completed all searches from the inception of each bibliographic database until November 1 and December 31, 2022. H.J. first searched the trial registries on November 2, 2022. Updates to searches in all bibliographic databases and trial registers were conducted on December 6, 2023. The tables in Appendix 1 outline the details of search histories for the bibliographic databases.

In a supplementary search, H.J. scrutinized the cited literature in all systematic reviews and meta-analyses from the list of retrieved articles, studies included in our review, and a Cochrane review by Buchbinder et al. [6] to identify further relevant articles to be entered into the screening and reviewing process.

Eligibility Criteria

This systematic review includes full-text publications reporting results from RCTs and observational studies (cohort and case/control studies). We excluded technical notes, review papers, meta-analyses, case reports, expert opinions, economic analyses, and publications with incomplete database information. When studies reported results unsuitable for analyses, the authors were contacted to ask for results in a format that would enable inclusion.

We included studies only on percutaneous procedures (not open procedures). Studies were included if the intervention included combined bone cement and a vertebral expander, such as Osseofix®, Spine Jack, SKy Bone Expander, intravertebral expandable pillar, vertebral body stenting, or similar products.

We excluded studies on vertebral body augmentation in combination with spinal fusion or instrumentation (such as pedicle screws or intervertebral cages), as well as augmentation using fillers other than bone cement. Studies on cadaveric spines, model organisms, prophylactic vertebral augmentation, and sacroplasty were excluded.

Eligible study populations consisted of persons with osteoporosis who had vertebral compression fractures (VCFs) and experienced pain from their fractures. Therefore, patients included in each study should meet these inclusion criteria: a vertebral fracture is present (no specific compression ratio), and pain from a fractured vertebra is present (no specific time period).

Publications or treatment groups were excluded from the review if they reported on vertebral augmentation in patients with the following conditions: spinal infection, spinal deformity not secondary to osteoporosis, primary tumors or metastases in the spine, or spinal re-fractures after previous augmentation.

As pain from the fractured vertebra(e) is the primary indication for treatment with vertebral body augmentation, we also included studies that did not specifically mention pain from the fracture(s). Likewise, we assumed that vertebral augmentation was not offered to patients with spinal deformities unrelated to osteoporosis unless specifically noted. Studies that did not specifically mention how patients were diagnosed with osteoporosis were accepted, as low-energy vertebral fractures are an indication of osteoporosis.

If a study included eligible and ineligible patients in the same cohort, we asked the authors if they would share their raw, de-identified data so that we could extract data for eligible patients only. If a study reported results for cohorts of all eligible patients, we included data for patients in these cohorts in our data synthesis.

Articles and trial reports in English, German, Norwegian, Swedish, and Danish were included, allowing the reviewers to conduct full-text appraisals of eligible articles.

Screening and Study Selection Process

All articles retrieved from searches in the five databases were exported to EndNote (Clarivate, Philadelphia, PA, USA), merged, and duplicates removed. The resulting publications were exported to Covidence (Melbourne, Australia), where additional duplicates were removed.

H.J. screened all titles and abstracts. L.C., L.W., and B.K. shared the role of independent second reviewer of titles and abstracts, and M.A. resolved any conflicts. L.C. and M.A. independently assessed full texts for potential inclusion and solved consensus-based disputes. Study characteristics were registered in two templates; one contains information reported in Tables 1-2, and the other contains additional details reported in Appendix 2. Reasons for excluding full-text articles were (1) the intervention was not relevant, (2) the outcomes were not relevant or the data format did not allow extraction and analysis, or (3) the study population was not relevant (Appendix 3).

**Table 1:** Study characteristics for included articles reporting on the effect of vertebral body augmentation on sagittal alignment outcomes PVP: percutaneous vertebroplasty, KP: kyphoplasty, MT: main thoracic (T1 to T9), TL: thoracolumbar (T10 to L2), LU: lumbar (L3 to L5), MRI: magnetic resonance imaging, NA: not available, AVF: adjacent vertebral fracture

Authors	Year	Study design	Inclusion period	Intervention	Edema on MRI	Symptom duration	n	Male-to-female ratio	Mean age	Mean follow-up (months)
Cao et al. [21]	2020	Longitudinal observational cohort study	2013-2018	KP	Yes	NA	Overall: 90	Overall: 20:70	69.3	0.1
							MT: 9	MT: 3:6		
							TL: 71	TL: 14:57		
							LU: 10	LU: 3:7		
Erkan et al. [22]	2009	Longitudinal comparative cohort study, prospective	2006-2007	KP	Yes		Overall: 28	Overall: 8:20		18
						Acute: <2 months	Acute: 15	Acute: 4:11	Acute: 70	
						Chronic: NA	Chronic: 13	Chronic: 4:9	Chronic: 74	
Kanayama et al. [23]	2015	Longitudinal observational cohort study	NA	KP	No	3.4 months	Overall: 56	7:49	75.3	32
Kim et al. [24]	2022	Longitudinal observational cohort study	2010-2017	PVP	NA	NA	No recollapse: 106	23:83	77.5	24
Oishi et al. [25]	2020	Longitudinal observational cohort study	2012-2015	KP	NA	4.2 months	No AVF: 23	4:19	80.8	24
Pumberger et al. [26]	2020	Longitudinal observational cohort study	2014-2018	KP	NA	4.7 weeks	Overall: 73	26:47	70	0.1
Su et al. [27]	2022	Longitudinal observational cohort study	2020	PVP	NA	NA	Overall: 42	8:34	80.74	3
Sutipornpalangkul et al. [28]	2016	Longitudinal observational cohort study	2007-2014	KP	Yes	NA	KP: 17	6:11	78.29	0.25
Yokoyama et al. [29]	2015	Longitudinal observational cohort study	2013-2014	KP	Yes	NA	Overall: 21	NA	77.1	1

**Table 2:** Study characteristics for included articles reporting on the effect of vertebral body augmentation on pulmonary function outcomes PVP: percutaneous vertebroplasty, KP: kyphoplasty, TH: thoracic (T8 to T11), TL: thoracolumbar (T12 to L2), LU: lumbar (L3 to L4), MRI: magnetic resonance imaging, NA: not available

Authors	Year	Study design	Inclusion period	Intervention	Edema on MRI	Symptom duration	n	Male-to-female ratio	Mean age	Mean follow-up (months)
Dong et al. [30]	2009	Non-randomized clinical trial	2006-2008		NA	NA	Overall: 38	Overall: 0:38		3
				PVP			PVP: 18		PVP: 70.2	
				KP			KP: 20		KP: 69.5	
Greven et al. [31]	2017	Longitudinal observational cohort study	NA	KP	NA	NA	Overall: 25	Overall: 11:14	70.4	1
							TH: 4			
							TL: 16			
							LU: 5			
Lee et al. [32]	2011	Longitudinal observational cohort study	2005-2006	PVP	NA	Acute	Overall: 72	10:62	75.7	3
Masala et al. [33]	2014	Longitudinal observational cohort study	NA	PVP	Yes	<3 months	Overall: 45	49:16	71.4	12
Sheng et al. [34]	2015	Longitudinal observational cohort study	NA	KP	NA	NA	Overall: 31	NA	71.2	3
Tanigawa et al. [35]	2008	Longitudinal observational cohort study	2005	PVP	NA	NA	Overall: 41	2:39	72.0	1
Wu et al. [36]	2018	Non-randomised clinical trial	2013-2015		Yes	NA	Overall: 69	Overall: 10:51	71.5	12
				KP			KP: 31	KP: 6:25		
				PVP			PVP: 30	PVP: 4:26		
Yang et al. [37]	2007	Longitudinal observational cohort study	NA	KP	NA	NA	Overall: 30	0:30	69.1	1

Outcomes and Data Extraction

**Figure 1:**
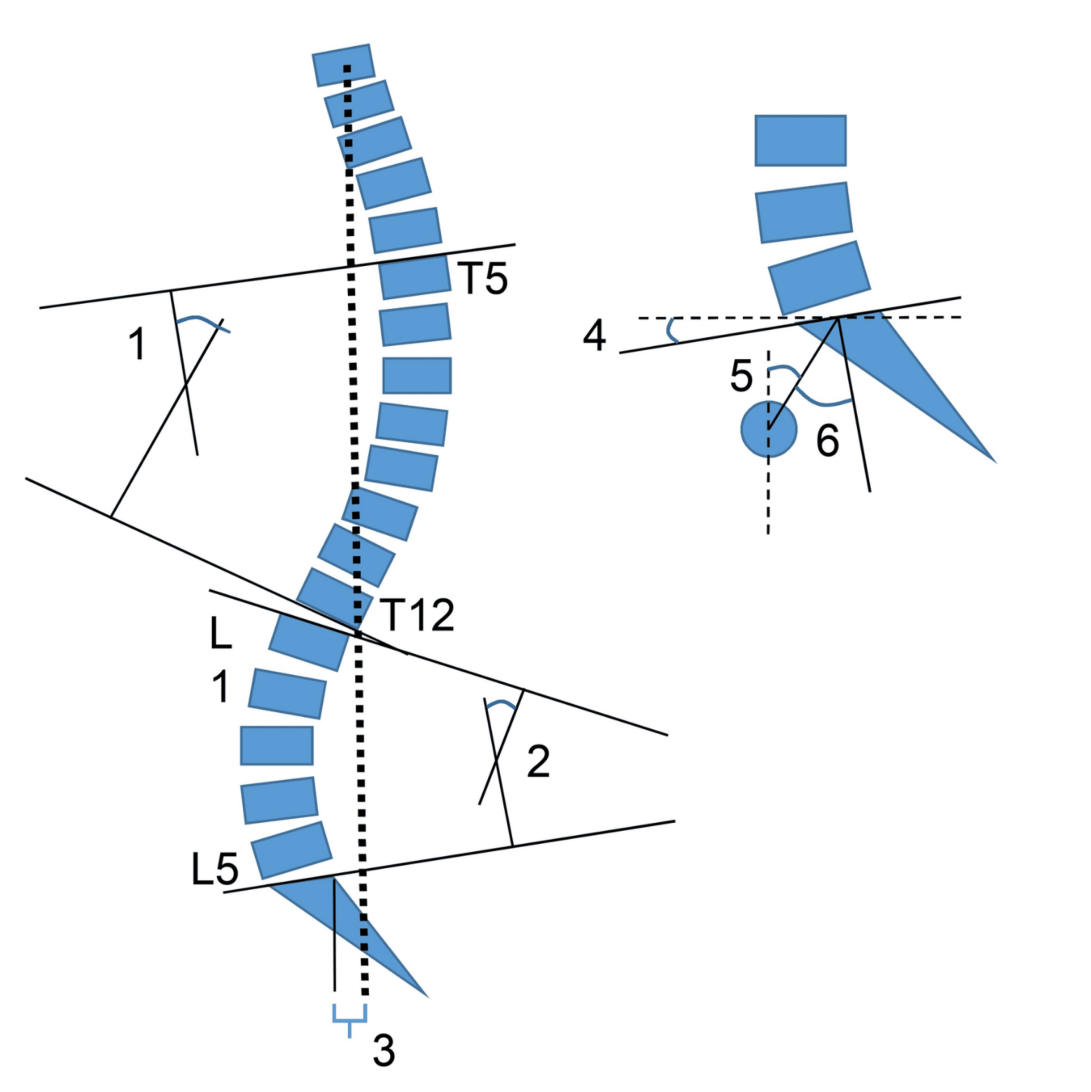
Sagittal alignment outcomes in included articles Outcomes were measured on standing radiographs. Results from each study are presented in Table 3. TK: thoracic kyphosis, LL: lumbar lordosis, SVA: sagittal vertical axis: distance between reference and C7 plumb line, SS: sacral slope, PT: pelvic tilt, PI: pelvic incidence

L.C. manually extracted and entered the data into Microsoft Excel (Microsoft Corp., Redmond, WA, USA). H.J. verified the extracted data. Initially, we extracted data for all sagittal alignment and pulmonary function outcomes to get an overview of available data (Tables 3-4). The key measure of spinal sagittal alignment is the sagittal vertical axis (SVA [38]), and the postoperative change in SVA is the primary outcome in this study. We also included other radiographic spinopelvic measures that allowed comparisons of patients’ sagittal alignment before and after vertebral body augmentation (Figure 1): pelvic incidence (PI) [39], sacral slope (SS) [40], pelvic tilt (PT) [40], thoracic kyphosis (TK) [38], lumbar lordosis (LL) [38], spino-sacral angle (SSA) [41], T1 pelvic angle (TPA) [42], and PI-LL [43].

**Table 3:** Input data for sagittal alignment outcomes in all relevant cohorts Due to variation in follow-up time points, results from one day postoperatively to the last follow-up were combined into AnyPost. SVA was the most commonly selected effect measure in the included studies. PVP: percutaneous vertebroplasty, KP: kyphoplasty, BI: Barthel index, SVA: sagittal vertical axis, ODI: Oswestry Disability Index, OVCF: osteoporotic vertebral compression fracture, VAS: Visual Analogue Scale, %diff: percentage difference, SSA: spino-sacral angle, TPA: T1 pelvic angle, PI: pelvic incidence, PI-LL: pelvic incidence minus lumbar lordosis, SS: sacral slope, PT: pelvic tilt, LL: lumbar lordosis, TK: thoracic kyphosis, KP: kyphoplasty, MT: main thoracic, TL: thoracolumbar, LU: lumbar, AVF: adjacent vertebral fracture, Pre: preoperatively, AnyPost: any time postoperatively, Post6wk: six weeks postoperatively, Post3mo: three months postoperatively, Post6mo: six months postoperatively, Post12mo: 12 months postoperatively, Post18mo: 18 months postoperatively

Author	Cao et al. [21]	Cao et al. [21]	Cao et al. [21]	Cao et al. [21]	Erkan et al. [22]	Erkan et al. [22]	Kanayama et al. [23]	Kim et al. [24]	Oishi et al. [25]	Pumberger et al. [26]	Su et al. [27]	Sutipornpalangkul et al. [28]	Yokoyama et al. [29]
Year	2020	2020	2020	2020	2009	2009	2015	2022	2020	2022	2022	2016	2015
Inclusion period	2013-2018	2013-2018	2013-2018	2013-2018	2006-2007	2006-2007	-	2010-2017	2012-2015	2014-2018	2020	2007-2014	2013-2014
Intervention group	KP_ MT	KP_ TL	KP_ LU	KP_ All	KP_ Acute	KP_ Chronic	KP	PVP_ NoRe collapse	KP_AVF	KP	PVP	KP	KP
N	9	71	10	90	15	13	56	106	23	73	42	17	21
Mean age	69.6	68.8	72.1	69.3	70	74	75.3	77.5	80.8	70	80.7	78.3	77.1
Mean follow-up (months)	0.1	0.1	0.1	0.1	18	18	32	24	24	0.1	3	0.25	1
Pre_ODI	-	-	-	-	86.0	86.0	-	-	-	-	83.1	-	-
AnyPost_ODI	-	-	-	-	54.0	44.0	-	-	-	-	21.2	-	-
PreOVCF_BI	-	-	-	-	-	-	-	-	-	-	79.5	-	-
Pre_BI	-	-	-	-	-	-	-	-	-	-	57.4	-	-
AnyPost_BI	-	-	-	-	-	-	-	-	-	-	96.5	-	-
Pre_VAS	-	-	-	-	-	-	55.0	-	-	37.0	-	-	79.8
AnyPost_VAS	-	-	-	-	-	-	20.0	-	-	17.0	-	-	23.8
Post6wk_VAS_%diff	-	-	-	-	59.0	48.0	-	-	-	-	-	-	-
Post3mo_VAS_%diff	-	-	-	-	50.0	46.0	-	-	-	-	-	-	-
Post6mo_VAS_%diff	-	-	-	-	47.0	43.0	-	-	-	-	-	-	-
Post12mo_VAS_%diff	-	-	-	-	44.0	41.0	-	-	-	-	-	-	-
Post18mo_VAS_%diff	-	-	-	-	42.0	37.0	-		-	-	-	-	-
Pre_SVA (mm)	-	-	-	14.5	83.0	97.0	31.0	21.0	76.8	50.8	49.0	-	70
AnyPostSVA	-	-	-	5.8	74.0	86.0	59.0	22.0	73.7	40.5	37.8	-	50.2
Post12mo_SVA	-	-	-	-	74.0	86.0	-	22.0	84.7	-	-	-	-
Pre_SSA	-	-	-	119.2	-	-	-	-	-	-	114.1	-	106.2
AnyPost_SSA	-	-	-	121.7	-	-	-	-	-	-	114.9	-	111.5
Pre_TPA	-	-	-	18.7	-	-	-	-	-	-	23.0	-	-
AnyPost_TPA	-	-	-	16.7	-	-	-	-	-	-	20.7		-
Pre_PI	-	-	-	52.2	-	-	-	-	-	56.1	-	-	54.6
AnyPost_PI	-	-	-	-	-	-		-	-	55.6	-	-	54.9
Pre_PI-LL	-	-	-	9.3	-	-	-	-	-	-	-	-	-
AnyPost_PI-LL	-	-	-	3.7	-	-	-	-	-	-	-	-	-
Pre_SS	-	-	-	30.0	-	-	-	-	-	35.9	-	-	28.8
AnyPost_SS	-	-	-	31.7	-	-	-	-	-	35.9	-	-	27.2
Pre_PT	-	-	-	24.0	-	-	-	-	-	20.7	-	-	25.3
AnyPost_PT	-	-	-	22.1	-	-	-	-	-	20.9	-	-	26.0
Pre_LL	-	-	-	48.4	-	-	-	-	-	33.2	36.0	-	41.8
AnyPost_LL	-	-	-	50.2	-	-	-	-	-	34.7	37.9	-	44.7
Pre_TK	-	-	-	37.8	-	-	-	21.7	-	46.8	25.8	-	-
AnyPost_TK	-	-	-	36.7	-	-	-	23.1	-	46.1	14.9	-	-

**Table 4:** Input data for pulmonary function outcomes in all relevant cohorts Due to variation in follow-up time points, results from day 1 postoperatively to the last follow-up were combined into a single category, AnyPost. When available, separate results for 12 months postoperatively were included. FEV1% was the most commonly selected effect measure in the included studies. PVP: percutaneous vertebroplasty, KP: kyphoplasty, FEV1%: percentage of predicted forced expiratory volume in one second, VAS: Visual Analogue Scale, ODI: Oswestry Disability Index, VC: vital capacity, TLC: total lung capacity, FVC: forced vital capacity, MVV: maximum voluntary ventilation, VC%: percentage of predicted vital capacity, FVC%: percentage of predicted forced capacity, Pre: preoperatively, AnyPost: any time postoperatively, Post12mo: 12 months postoperatively, Post1mo: one month postoperatively

Authors	Dong et al. [30]	Greven et al. [31]	Greven et al. [31]	Greven et al. [31]	Greven et al. [31]	Lee et al. [32]	Masala et al. [33]	Sheng et al. [34]	Tanigawa et al. [35]	Wu et al. [36]	Wu et al. [36]	Yang et al. [37]
Year	2009	2017	2017	2017	2017	2011	2014	2015	2008	2018	2018	2007
Inclusion period	2006-2008	-	-	-	-	2005-2006	2011-2013	-	2005	2013-2015	2013-2015	-
Intervention	PVP	KP_Th8-11	KP_Th12-L2	KP_L3-4	KP_All	PVP	PVP	KP	PVP	PVP	KP	KP
N	18	4	16	5	25	72	45	31	41	30	31	30
Mean age	70.2	-	-	-	70.4	75.7	71.4	71.2	72.0	73.6	71.5	69.1
Mean follow-up (mo)	3	1	1	1	1	3	12	3	1	12	12	1
Pre_VAS	83.0	77.4	78.5	62.4	75.1	79.0	77.0	80.0	70.0	-	-	-
AnyPost_VAS	29.0	15.6	0.0	15.8	5.7	17.0	22.0	23.0	19.0	-	-	-
Post12mo_VAS	-	-	-	-	-	-	2.1	-	-	-	-	-
Pre_ODI	-	73.4	66.5	53.3	65.0	-	-	-	-	-	-	-
Post1mo_ODI	-	18.5	2.5	18.2	8.2	-	-	-	-	-	-	-
Pre_VC	2.4	-	-	-	-	-	-	-	-	2.3	2.2	2.4
AnyPost_VC	2.4	-	-	-	-	-	-	-	-	2.5	2.5	2.5
Pre_VC	-	-	-	-	-	-	-	-	-	2.3	2.2	-
Post12mo_VC	-	-	-	-	-	-	-	-	-	2.5	2.5	
Pre_TLC	4.1	-	-	-	-	-	-	-		-	-	4.0
AnyPost_TLC	4.1	-	-	-	-	-	-	-	-	-	-	4.0
Pre_FVC	2.2	-	-	-	-	-	-	-	-	2.2	2.1	2.2
AnyPost_FVC	2.4	-	-	-	-	-	-	-	-	2.3	2.3	2.3
Pre_FVC	-	-	-	-	-	-	-	-	-	2.2	2.1	-
Post12mo_FVC	-	-	-	-	-	-	-	-	-	2.3	2.3	
Pre_MVV	57.7	-	-	-	-	-	-	-	-	54.4	54.2	57.6
AnyPost_MVV	60.7	-	-	-	-	-	-	-	-	63.2	62.7	60.7
Post12mo_MVV	-	-	-	-	-	-	-	-	-	63.2	62.7	-
Pre_VC%	-	-	-	-	-	-	81.5	-	-	-	-	-
Post12mo_VC%	-	-	-	-	-	-	98.6	-	-	-	-	-
Pre_FEV1%	-	73.0	71.0	72.0	71.5	58.3	97.4	60.2	77.0	62.2	61.0	-
AnyPost_FEV1%	-	91.0	86.0	86.0	86.8	68.0	97.4	60.8	77.6	63.5	61.6	-
Pre_FEV1%	-	-	-	-	-	-	97.4	-	77.0	62.2	61.0	-
Post12mo_FEV1%	-	-	-	-	-	-	97.4	-	77.6	63.5	61.6	-
Pre_FVC%	-	-	-	-	-	58.0	77.8	74.3	85.2	-	-	-
AnyPost_FVC%	-	-	-	-	-	76.0	98.2	84.9	91.5	-	-	-
Pre_FVC%	-	-	-	-	-	-	77.8	-	85.2	-	-	-
Post12mo_FVC%	-	-	-	-	-	-	98.2	-	91.5	-	-	-

We identified six studies that reported on sagittal alignment outcomes for participants with osteoporosis but no VCFs [11,44-48]. By comparing results from the included study populations to those for elderly osteoporosis patients without VCFs, we could gauge the severity of malalignment in the included study populations.

For the pulmonary outcomes, the following data points were collected: vital capacity (VC) [49], total lung capacity (TLC) [49], forced vital capacity (FVC) [49], maximum voluntary ventilation (MVV) [50], percentage of predicted vital capacity (VC%), percentage of predicted forced expiratory volume in 1 second (FEV1%), and percentage of predicted forced capacity (FVC%) (predictions according to the American Thoracic Society [51]).

Patient-reported outcomes, including the Oswestry Disability Index (ODI) [52] and pain scores (Visual Analogue Scale (VAS)) [53], were collected when available.

Risk-of-Bias Assessment

M.A. and H.J. independently assessed the risk of bias (RoB) for each study using the quality assessment tool for quantitative studies by the Effective Public Health Practice Project (EPHPP) [54]. The EPHPP tool can be applied to randomized and observational studies, assessing potential bias due to six study characteristics: selection of study population, study design, confounders, incomplete blinding of assessors and participants, data collection methods, and withdrawals and dropouts. M.A. and H.J. agreed to assess each category of potential bias and discuss any discrepancies. Results from the RoB assessment are reported using the online RobVis visualization tool (McGuinness LA and Higgin JP, Bristol, UK).

Data Synthesis

A weighted average was calculated for each of the outcomes of interest. A sub-analysis excluding cases with a high RoB was also performed. We did not perform a meta-analysis due to the considerable heterogeneity among the included studies. The differences in study designs, populations, interventions, and outcomes prevented meaningful data aggregation.

Results​​​​​​

**Figure 2:**
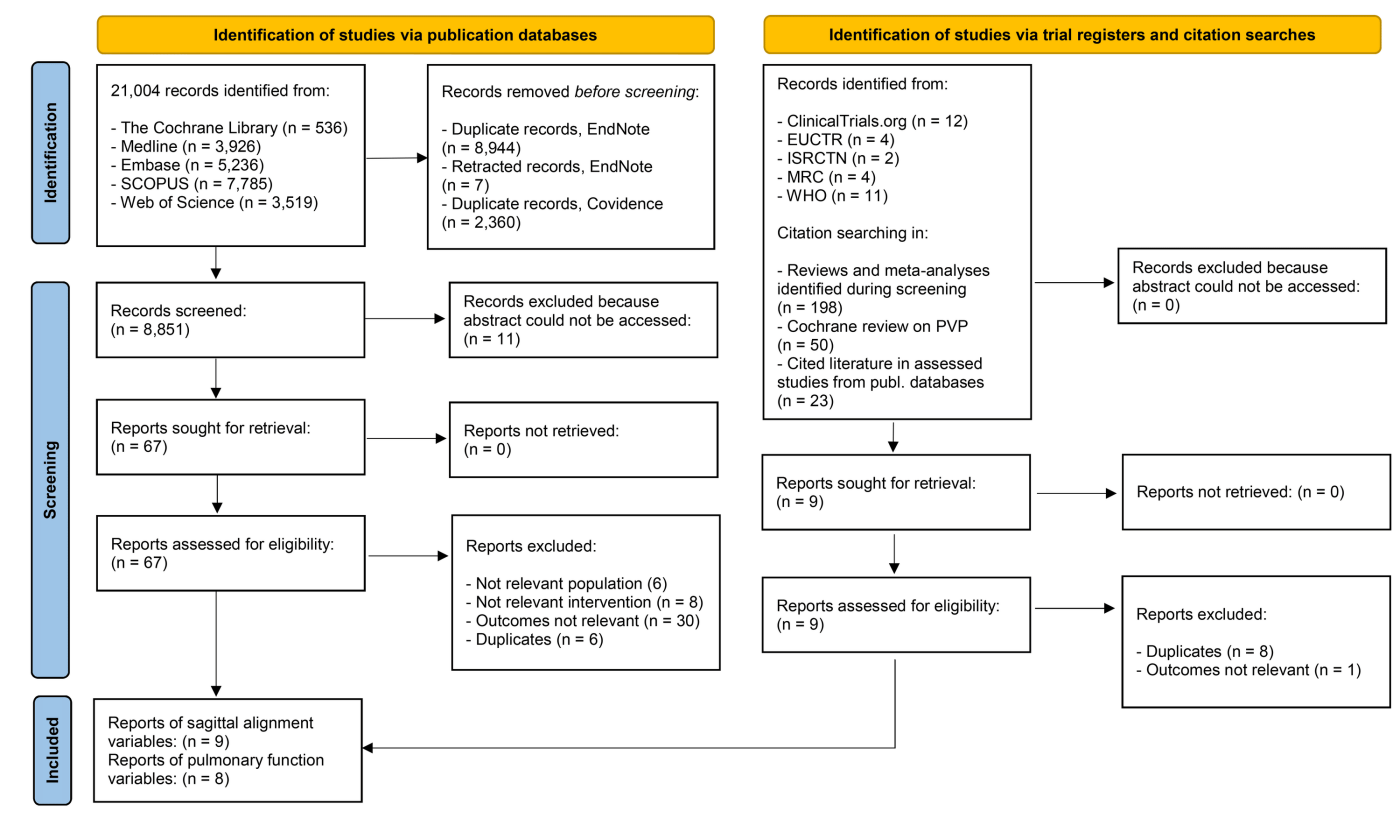
PRISMA diagram presenting the identification and screening process On the left are publication databases, and on the right are records from trial registries and citation searches. We applied a multi-stranded search strategy, and the diagram shows the overall number of records. PRISMA: Preferred Reporting Items for Systematic Reviews and Meta-Analyses, EUCTR: EU Clinical Trials Register, ISRCTN: International Standard Randomised Controlled Trial Number, MRC: Medical Research Council, WHO: World Health Organization

A total of 21,004 records were identified from bibliographic databases. After removing duplicates, 8,851 records were screened, and 67 were included in the full-text analysis. In addition, 33 records from trial registries and 271 cited references were screened. Data from nine articles reporting on sagittal alignment for 12 eligible cohorts [21-29] were included in the analysis. Also included were data from eight articles reporting on pulmonary function outcomes for 12 eligible cohorts [30-37]. An overview of the selection process is presented in a PRISMA flow diagram (Figure 2) [20].

Tables 1-2 present study characteristics for all included articles (additional details in Appendix 2). The included articles comprise reports on two non-randomized clinical studies, a prospective longitudinal comparative cohort study, and 14 longitudinal observational cohort studies. Studies excluded during full-text review and reasons for exclusion are presented in Appendix 3.

**Figure 3:**
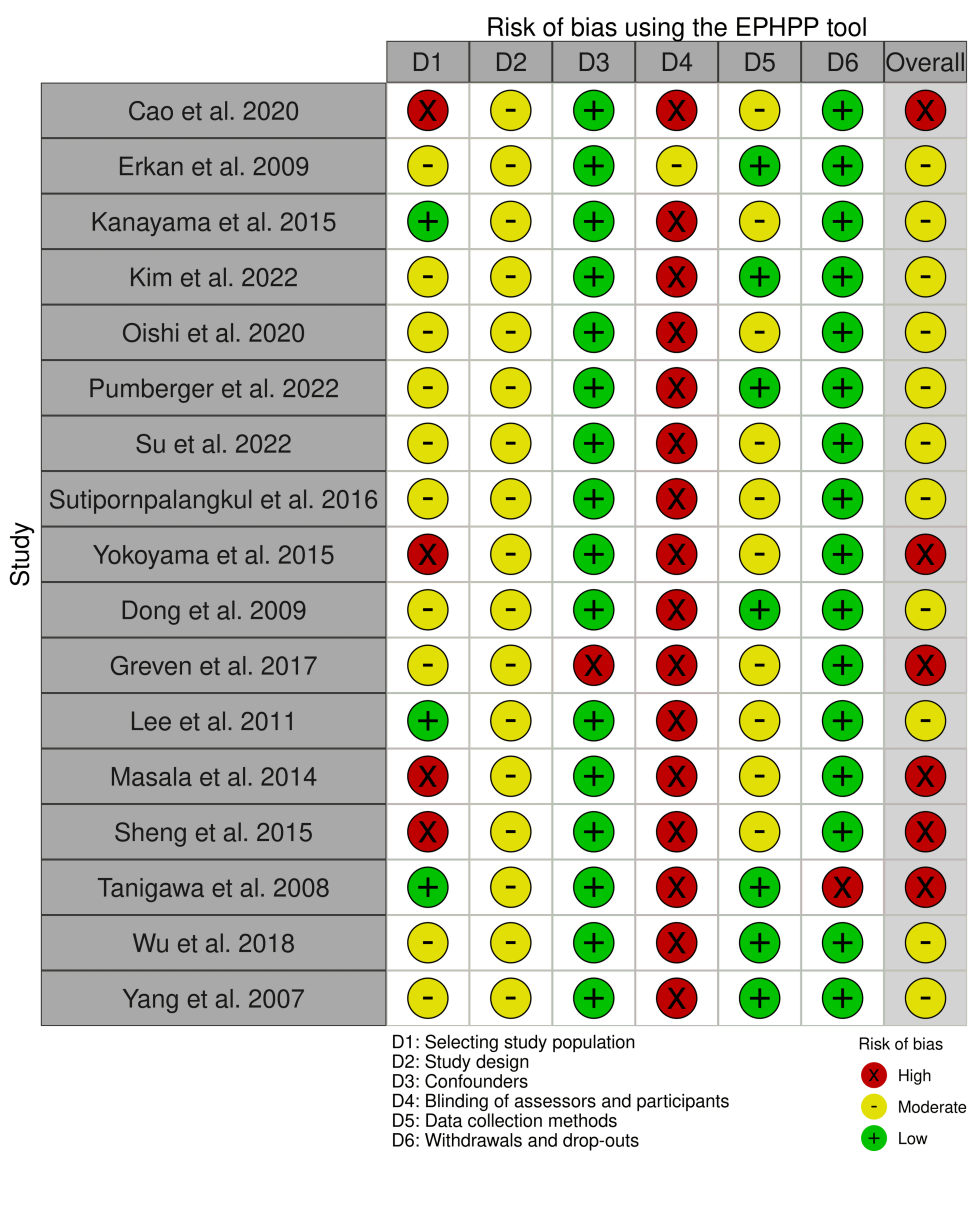
Results of the RoB assessment For the assessment, we used the quality assessment tool for quantitative studies by the EPHPP [54]. EPHPP: Effective Public Health Practice Project, RoB: risk of bias

Strong, moderate, and weak study quality ratings were converted into low, moderate, or high RoB assessments, as shown in Figure 3. We did not include any RCTs, which is reflected in the moderate RoB, mainly due to the study design and the high RoB due to a lack of blinding. Overall, the RoB due to confounders and dropouts was low.

Data on sagittal alignment measures were extracted for 456 individuals (male-to-female ratio 102:333, excluding Yokoyama et al. [29], who did not report sex ratio) with mean reported ages ranging from 69.3 to 80.8 years (Table 3). Data on pulmonary function measures were extracted for 343 individuals (male-to-female ratio 68:275) with mean reported ages ranging from 69.1 to 75.7 years (Table 4). Follow-up time points varied widely between studies. To handle this, we combined time points from one day to the last postoperative follow-up for data analysis. This resulted in data for three time points: preoperatively, any time postoperatively (Tables 5-6), and 12 months postoperatively for VC% (Table 6).

**Table 5:** Weighted mean effect of vertebral augmentation on sagittal alignment measures Results are presented for all studies with medium-to-high RoB and a sub-analysis excluding cases with a high RoB. For comparison, results are shown on the right for study populations with osteoporosis but no OVCF. Except for PI-LL, all mean outcome measures from study cohorts fall within the ranges reported for persons with no OVCF. Vertebral augmentation did not affect any of the outcomes. ^a^ Data point available for only one article (Erkan et al. [22]), ^b^ Data point not available for medium RoB articles ODI: Oswestry Disability Index, VAS: Visual Analogue Scale, SVA: sagittal vertical axis, SSA: spino-sacral angle, TPA: T1-pelvic angle, PI-LL: pelvic incidence minus lumbar lordosis, SS: sacral slope, PT: pelvic tilt, LL: lumbar lordosis, TK: thoracic kyphosis, RoB: risk of bias, SD: standard deviation, VCF: vertebral compression fracture, OVCF: osteoporotic vertebral compression fracture, NR: not reported, NA: not available

	Medium to high RoB		Medium RoB		Literature, patients with osteoporosis but no VCF	
	Number of cohorts	Weighted mean (SD)	Number of cohorts	Weighted mean (SD)	Number of cohorts (references)	Range of means
Number of studies		9		7	-	-
Number of cohorts		12		8	-	-
N		456		345	-	-
Males, %	11	35%	8	24%	-	-
Age, years	12	75.53 (4.39)	8	75.75 (4.30)	-	-
ODI	1		1			
Preoperative*		84.26 (NR)		84.26 (NR)	-	-
Any postoperative^a^		32.46 (NR)		32.46 (NR)	-	-
VAS (1-10)	7		3			
Preoperative		5.80 (1.55)		5.36 (2.09)	-	-
Any postoperative		2.12 (0.31)		1.09 (0.22)	-	-
SVA (mm)	12		8			
Preoperative		39.94 (28.56)		43.42 (25.99)	6 (11,44-48)	-15.5–63.6
Any postoperative		34.05 (26.73)		38.76 (23.38)	-	-
SSA (^o^)	4		2			
Preoperative		116.30 (6.73)		115.36 (3.12)	2 (11,47)	114.9–124.8
Any postoperative		118.73 (6.64)		116.31 (3.37)	-	-
TPA (^o^)	3		1			
Preoperative		20.10 (5.07)		22.98 (8.38)	-	-
Any postoperative		18.07 (3.96)		20.69 (7.37)	-	-
PI-LL (^o^)	3		0			
Preoperative^b^		6.33 (7.90)		NA	2 (44,48)	11.6–4.7
Any postoperative^b^		2.98 (5.70)		NA	-	-
SS (^o^)	6		2			
Preoperative		32.27 (3.04)		35.31 (2.28)	5 (11,45-48)	25.1–35.7
Any postoperative		32.64 (3.80)		34.89 (3.83)	-	-
PT (^o^)	6		2			
Preoperative		23.28 (4.12)		22.13 (5.51)	6 (11,44-48)	16.3–27.5
Any postoperative		22.41 (4.01)		21.88 (3.85)	-	-
LL (^o^)	3		0			
Preoperative^b^		41.31 (9,78)		NA	5 (11,45-48)	27.3–46.7
Any postoperative^b^		43.25 (3.50)		NA	-	-
TK (^o^)	7		3			
Preoperative		32.80 (9.80)		30.78 (13.46)	5 (11,45-48)	30.9–45.3
Any postoperative		29.34 (11.26)		29.13 (16.16)	-	-

**Table 6:** Weighted mean effect of vertebral augmentation on pulmonary function measures Results are presented for all studies with medium-to-high RoB and a sub-analysis excluding cases with a high RoB. A positive effect was seen in outcomes reported as a percentage of the expected value. ^a^ values are from studies with medium RoB only, ^b^ Masala et al. [33], ^c^ Lee et al. [32] ODI: Oswestry Disability Index, VAS: Visual Analogue Scale, VC: vital capacity, TLC: total lung capacity, FVC: forced vital capacity, MVV: maximum voluntary ventilation, VC%: percent vital capacity, FEV1%: percent forced expiratory volume in one second, FVC%: percent forced vital capacity, RoB: risk of bias, NA: not available

	Medium to high RoB		Medium RoB	
	Number of cohorts	Weighted mean (SD)	Number of cohorts	Weighted mean (SD)
Number of studies		8		4
Number of cohorts		12		6
N		343		201
Males		20%		10%
Age, years		71.9 (31.4)		73.0 (28.0)
ODI	3		0	
Preoperative		65.0 (10.2)		NA
Any postoperative		22.6 (3.8)		NA
VAS (1-10)	9		3	
Preoperative		7.8 (0.6)		8.2 (0.2)
Any postoperative		2.1 (0.6)		2.3 (0.7)
VC (L)	5		5	
Preoperative^a^		2.4 (0.1)		2.4 (0.1)
Any postoperative^a^		2.5 (0.0)		2.5 (0.0)
TLC (L)	3		3	
Preoperative^a^		4.1 (0.0)		4.1 (0.0)
Any postoperative^a^		4.1 (0.0)		4.1 (0.0)
FVC (L)	5		5	
Preoperative^a^		2.2 (0.1)		2.2 (0.1)
Any postoperative^a^		2.3 (0.0)		2.3 (0.0)
MVV (L)	5		5	
Preoperative^a^		56.5 (2.0)		56.5 (2.0)
Any postoperative^a^		62.3 (1.2)		62.3 (1.2)
VC% (% of predicted)^b^	1		1	
Preoperative		81.5 (3.9)		81.5 (3.9)
12-month postoperative		98.6 (1.2)		98.6 (1.2)
FEV1% (% of predicted)	9		3	
Preoperative^a^		69.6 (12.2)		59.8 (2.0)
Any postoperative^a^		73.9 (13.9)		65.5 (3.3)
FVC% (% of predicted)	4		1	
Preoperative		71.3 (11.5)		58.0 (23.8)^c^
Any postoperative		86.1 (9.1)		76.0 (22.1)^c^

We analyzed data extracted for all sagittal alignment outcomes except the Barthel index, which was only reported by Su et al. (Table 3) [27]. It was not possible to make comparisons of the pulmonary function outcomes, FEV1%/FVC% ratio, inspiratory capacity (IC), residual volume (RV), functional residual capacity (FRC), and peak respiratory flow (PEF). The FEV1%/FVC% ratio was included in two studies, but the values varied widely [32,34]. PEF [31] and IC, RV, and FRC [37] were included in only one study.

None of the sagittal alignment parameters in our analysis improved after vertebral augmentation (Table 5). SVA, the primary outcome in our analysis, and PI-LL had very high standard deviations pre- and postoperatively. The weighted mean values for all preoperatively sagittal alignment parameters in the study populations were within the range reported in the literature for patients with osteoporosis but no VCFs.

Unadjusted pulmonary function measures (VC, TLC, FVC, MVV) did not improve after vertebral augmentation (Table 6). When the percentage of predicted values was analyzed, vertebral augmentation positively affected pulmonary function (VC%, FVC%, FEV1%). Results for VC% were based on one cohort with a moderate RoB, and results for FVC% were based on four cohorts, of which one had a moderate RoB. FEV1% was the most widely used measure, and the most distinct improvement after vertebral augmentation was seen when comparing weighted means of the three studies with a moderate RoB.

Data on perceived level of disability (ODI) and pain (VAS) were collected in studies on both sagittal alignment (ODI: two cohorts, moderate RoB; VAS: seven cohorts, three with a moderate RoB) (Table 5) and pulmonary function (ODI: three cohorts, high RoB; VAS: nine cohorts, three with a moderate RoB) (Table 6). Overall, vertebral augmentation had a positive effect on both ODI and VAS.

Discussion

The present study is the first to examine systematically collected literature on the effect of vertebral augmentation on global sagittal alignment and pulmonary function in patients with OVCFs. We found no significant impact of vertebral augmentation on spinal sagittal alignment parameters. However, it is equally noteworthy that there was no deterioration in the SVA or TK. Our analysis showed that vertebral augmentation does improve pulmonary function when measured as a percentage of expected values. Overall, vertebral augmentation also reduced experienced pain levels (VAS) and disability scores (ODI), as previously reported [55,56].

Spinal Sagittal Alignment

Vertebral augmentation of OVCFs did not significantly change global spinal alignment as measured by SVA, SSA, TPA, PI-LL, SS, PT, LL, and TK. This aligns with a meta-analysis by Najjar et al. [57] on four studies with no overall effect of kyphoplasty on sagittal alignment parameters in patients with OVCFs. The inability to identify changes in spinal sagittal alignment may partly be due to incomplete data, which, in turn, is due to variability in follow-up time points, inconsistency in measurements chosen by different authors (Tables 3-4), and differences in participant positioning for radiographs. In addition, the heterogeneous study populations, mainly due to the inclusion of both lumbar and thoracic fractures, influence the results.

The primary outcome in this study, the SVA [38], showed a modest 4.66 mm change from preoperatively to last follow-up in studies with a moderate RoB. Given the variability in SVA, this modest change may not be clinically relevant. Preoperative SVA in our analysis varied significantly, from 14.5 mm [21], within the normal range, to 97 mm [22], denoting malalignment.

Pulmonary Function

Building on previous work linking sagittal alignment with pulmonary function, the authors of the included studies hypothesized that improved sagittal alignment after vertebral augmentation of OVCFs would enhance pulmonary function. Vertebral augmentation mostly improved VC% (results from only one study [33]) and FVC%. Our review did not represent all measures from the included articles because data were too scarce for analysis.

A 2007 systematic review by Harrison et al. [14] cautiously concluded that increased kyphosis due to OVCFs correlated with a modest decline in VC. Tanigawa et al. [35] showed that improved pulmonary function (FVC%) was only seen when treating thoracic OVCFs, with no difference based on the number of OVCFs. In contrast, Morseth et al. [58] found a significant correlation between the number of VCFs and FVC% and FEV1% in men but not women. FVC% and FEV1% did not differ significantly between fracture sites (T4-T12, L1-L4) in either men or women.

Horie et al. [59] reported a correlation between LL and reduced pulmonary function in healthy women with an average age of 76.8 years. Rahman et al. [60] found that FEV1% and FVC% were significantly negatively correlated with TK, but the percentage predicted maximum inspiratory and expiratory pressure was significantly negatively correlated with LL.

One explanation for improving pulmonary function is that back pain due to TK restricts normal breathing [61]. Our analysis's considerable mean pain reduction may improve pulmonary function after vertebral augmentation. An alternative explanation is that kyphosis may decrease the anterior-posterior diameter of the thoracic cavity, and changes in thoracic curvature will affect ribs and intercostal muscle function [60]. Likewise, it has been suggested that increased LL may decrease diaphragm constriction efficiency and thereby lower FVC [59,60]. Of the studies included in our analysis, only thoracic VCFs were treated in two studies, while both thoracic and lumbar VCFs were treated in six. Therefore, our results suggest that the improvement in pulmonary function may be due to a combination of underlying physiological changes.

Limitations

Our analysis is based on two non-randomized clinical studies, a prospective longitudinal comparative cohort study, and 14 longitudinal observational cohort studies. The results from these studies are considered level II and level III evidence.

Follow-up time points and length varied widely among the included studies, and data were merged into “preoperative” and “any time postoperative” for all outcomes. Merging data from the entire follow-up period, one risks reducing the level of detail and allowing data from different time points to cancel each other out.

The number of participants in studies on pulmonary function is often low. In the included articles, cohorts ranged from 72 participants [32] to four to five participants [31]. Reference values for pulmonary function measures depend on several factors, such as age, sex, standing height, and ethnicity [62]. However, the included articles did not report the specific reference values used to calculate percentage predicted outcomes. In addition, only one study reported which height measure they used to estimate predicted values of pulmonary function [32]; using different height measures is a source of considerable variability in predicted measures of pulmonary function [14,63].

No studies reported the effect on sagittal alignment and pulmonary function when combining bone cement and intravertebral instrumentation. Our study did not include spinal sagittal alignment as measured by local kyphosis at the level of OVCFs or treated vertebral body height, since it has been extensively studied elsewhere [64-66].

Literature was identified using a multi-stranded search strategy to ensure that we did not miss any relevant articles that failed to mention in the title, abstract, and keywords that their study population consisted of participants with osteoporosis. Despite a thorough search and screening process, we may have missed relevant studies, particularly those published in Chinese.

## Conclusions

Vertebral augmentation of OVCFs did not affect sagittal alignment parameters, possibly due to the heterogeneity in reported outcomes, follow-up time points, and study populations. Vertebral augmentation positively affected pulmonary function measured as VC%, FVC%, and FEV1%. However, results for VC% and FVC% are based on a very limited number of studies. In addition, vertebral augmentation had a positive effect on experienced pain level and disability.

The data in this systematic review were heterogeneous, considerably restricting the data analysis. In addition, we have pointed out critical factors that have not been thoroughly reported in previous articles. We want to encourage the scientific community to agree on optimal follow-up time points and core spinopelvic parameters, accurate reporting on treated fracture levels, positioning instructions for imaging, and calculation of predicted pulmonary function values. Each clinical study would then contribute to a valuable compilation of data on the effect of vertebral augmentation procedures on global spinal sagittal alignment and pulmonary function in patients with OVCFs.

## Appendices

Appendix 1.1

Medline search history exemplifies bibliographic database searches. We applied a multi-stranded search strategy, and Table 7 represents searches for publications that mentioned osteoporosis in their title, abstract, or keywords. Database: Ovid MEDLINE(R) ALL.

**Table 7:** Medline search history exemplifying the bibliographic database searches with search terms related to osteoporosis

#	Search terms	Hits 1 Nov 2022 (including osteoporosis terms)
1	exp spine/	161,299
2	(spine or spinal or vertebra$).mp.	671,444
3	exp fractures, bone/	203,585
4	(fractur$ or compres$).mp.	515,957
5	exp Spinal Fractures/	17,075
6	1 or 2	682,507
7	3 or 4	518,239
8	exp Osteoporosis/	61,381
9	osteopor$.mp.	102,968
10	8 or 9	102,968
11	6 and 7 and 10	17,215
12	5 and 10	6,573
13	11 or 12	17,215
14	exp Bone Cements/	24,629
15	(bone adj3 cement$).mp.	16,794
16	(bone adj3 glue$).mp.	155
17	(bone adj3 past$).mp.	394
18	exp Fracture Fixation, Internal/	49,852
19	(facture adj3 fixat$).mp.	4
20	exp Cementoplasty/	3,385
21	cementoplast$.mp.	375
22	vertebroplast$.mp.	4,266
23	kyphoplast$.mp.	2,363
24	KIVA.mp.	80
25	skyphoplast$.mp.	4
26	(vertebral adj3 augmentation).mp.	825
27	(vertebral adj3 body adj3 augmentation).mp.	80
28	or/14-27	81,205
29	13 and 28	3,210

Appendix 1.2

Medline search history exemplifies bibliographic database searches. Table 8 represents bibliographic database searches for publications that did not mention osteoporosis in their title, abstract, or keywords. We added different combinations of search terms related to posture to focus the search. Database: Ovid MEDLINE(R) ALL.

**Table 8:** Medline search history exemplifying the bibliographic database searches with search terms related to posture

#	Search terms	Hits 24 Oct 2023 (including posture terms)
1	exp spine/	166,157
2	(spine or spinal or vertebra$).mp.	711,327
3	exp fractures, bone/	210,264
4	(fractur$ or compres$).mp.	560,934
5	exp Spinal Fractures/	17,899
6	1 or 2	722,721
7	3 or 4	563,273
8	6 and 7	85,050
9	5 or 8	85,050
10	exp Bone Cements/	25,304
11	(bone adj3 cement$).mp.	17,752
12	(bone adj3 glue$).mp.	165
13	(bone adj3 past$).mp.	418
14	exp Fracture Fixation, Internal/	51,850
15	(fracture adj3 fixat$).mp.	72,821
16	exp Cementoplasty/	3,567
17	cementoplast$.mp.	413
18	vertebroplast$.mp.	4,643
19	kyphoplast$.mp.	2,673
20	KIVA.mp.	86
21	skyphoplast$.mp.	4
22	(vertebral adj3 augmentation).mp.	922
23	(vertebral adj3 body adj3 augmentation).mp.	89
24	or/10-23	105,500
25	9 and 24	10,655
26	exp Postural Balance/	28,340
27	((sagittal or body or postur*) adj3 (balance or alignment or equlibrium)).mp	38,193
28	26 or 27	38,221
29	25 and 28	153

Appendix 2

Additional study characteristics are included in the data analysis and overview.

**Table 9:** Characteristics of all included studies OVCF: osteoporotic vertebral compression fracture, VCF: vertebral compression fracture, KP: kyphoplasty, PKP: percutaneous kyphoplasty, PVP: percutaneous vertebroplasty, RF-TVA: radiofrequency-targeted vertebral augmentation, MRI: magnetic resonance imaging, STIR: short tau inversion recovery, BI: Barthel index, SVA: sagittal vertical axis, SSA: spino-sacral angle, TPA: T1 pelvic angle, TK: thoracic kyphosis, LL: lumbar lordosis, PI: pelvic incidence, PT: pelvic tilt, SS: sacral slope, PI-LL: pelvic incidence minus lumbar lordosis, VAS: Visual Analogue Scale, ODI: Oswestry disability index, %FEV1: percent predicted forced expiratory volume in one second, %FVC: percent predicted forced vital capacity, FEV1/FVC: forced expiratory volume in one second to forced vital capacity, VC: vital capacity, TLC: total lung capacity, MVV: maximum voluntary ventilation, AVF: adjacent vertebral fracture

Publication	Title	Aim of study	Study setting	Study design	Study population	No. of participants	Treated compression fractures	Intervention	Inclusion criteria	Exclusion criteria	Follow-up time points	Relevant outcomes	Authors' main conclusion(s)
Cao et al. 2020 [21]	Percutaneous kyphoplasty for osteoporotic vertebral compression fractures improves spino-pelvic alignment and global sagittal balance maximally in the thoracolumbar region	To analyze the differences in sagittal balance parameters after a vertebral fracture at different sites and to analyze the differences in sagittal balance improvement after PKP at different fracture sites	Shandong Provincial Hospital, Jinan, China	Longitudinal observational study	Patients with OVCFs receiving treatment from 2013 to 2018. Age 67.9 ± 6.2 years. Subgroups according to site of fracture: MT group (T1 to T9), TL group (T10 to L2), and LU group (L3 to L5)	Overall: n=90 (70 W, 20 M). MT group: n=9 (6 W, 3 M), TL group: n=71 (57 W, 14 M), and LU group: N=10 (7 W, 3 M)	T1-T9, T10-L2, L3-L5, only single fracture: NA	KP	(1) The vertebral compression ratio of the injured vertebrae is <80%. (2) Osteoporosis confirmed via bone mineral density. (3) Fractured vertebrae showed a high signal intensity on STIR MRI images and a low signal intensity on T1-weighted MRI images. (4) Imaging data were complete	(1) Lumbar disc herniation, spondylolisthesis, scoliosis, spinal osteoarthritis, ankylosing spondylitis, spinal tumors, and spinal tuberculosis. (2) History of spinal surgery. (3) Hip and knee joint limitations. (4) Spinal cord compression with clinical manifestations of spinal cord and cauda equina nerve injury. (5) Pathogenic fracture caused by a tumor or an incomplete posterior wall of the vertebral body. (6) Patients could not stand upright independently or did not have a standing X-ray	Preoperative + 2-3 days postoperative	VAS TK, LL, PI, PT, SS, PI-LL, SVA, SSA, and TPA	PKP is an effective treatment for thoracolumbar (T10-L2) OVCFs. PKP can relieve the low back pain caused by fractures and correct the pelvic posterior rotation during sagittal compensatory balance within 2–3 days. PKP can significantly improve the angular parameters, TPA, SSA, and overall sagittal alignment.
Erkan et al. 2009 [22]	Does timing matter in performing kyphoplasty? Acute versus chronic compression fractures	To compare the clinical outcomes, the radiographic outcomes, and the complication rates of symptomatic acute (<10 weeks) and chronic (>16 weeks) osteoporotic VCFs treated with KP	Celal Bayar University School of Medicine, Manisa, Türkiye	Longitudinal comparative cohort study, prospective	Patients with symptomatic osteoporotic VCFs receiving treatment in 2006-2007. Age 72 (62-79) years	Overall: n=28 (20 W, 8 M). Acute group: n=15 (11 W, 4 M) and chronic group: n=13 (9 W, 4 M)	T11-L4, only single fracture: no	KP	(1) Severely disabling back pain, which was reproduced by deep palpation over the spinous process at the involved level. (2) VCFs not responsive to bed rest, activity modification, bracing, and analgesics for at least two weeks	(1) Subacute fractures. (2) Fractures not showing oedema on MRI	Preoperative + 6 weeks postoperative + 3, 6, 12, 18 months postoperative	ODI, VAS, SVA, cement leakage, and subsequent fractures	KP improves clinical and radiological outcomes in patients with acute (<10 weeks) or chronic (>16 weeks) VCFs. The outcomes are mostly better in the acute group, but not always significantly
Kanayama et al. 2015 [23]	Does balloon kyphoplasty improve the global spinal alignment in osteoporotic vertebral fracture?	To review radiographic and clinical outcomes of balloon KP for delayed union or non-union after osteoporotic vertebral fracture, and to evaluate its effect on the global spinal alignment	Hakodate Central General Hospital, Hokkaido, Japan	Longitudinal observational study	Patients with failed nonoperative treatment for OVCF. Treatment period not mentioned. Age 75.3 (49-71) years. SVA analysis in sub-groups: (1) sagittal imbalance (SVA <50 mm), (2) previous OVCF, and (3) no previous OVCF	Overall: n=56 (49 W, 7 M)	T6-L5, only single fracture: no	KP	NA	NA	Preoperative + immediately postoperative + final follow-up (8-91 months)	VAS, SVA, and subsequent fractures	KP contributed to immediate pain relief, but did not improve the global sagittal spinal alignment after OVCF
Kim et al. 2022 [24]	Correlation of sagittal imbalance and recollapse after percutaneous vertebroplasty for thoracolumbar osteoporotic vertebral compression fracture: A multivariate study of risk factors	To evaluate the risk factors for recollapse following PVP in patients with thoracolumbar OVF and the association between recollapse and sagittal spinal parameters	Daejeon Eulji Medical Center, Daejeon, Korea	Longitudinal observational study	Patients who underwent single-level PVP 2010-2017. Age 77.5 ± 8.6 years	No recollapse group: n=106 (83 W, 23 M)	Thoracolumbar, only single fracture: yes	Vertebroplasty	(1) Ambulatory with senile or postmenopausal osteoporosis. (2) Recent OVF diagnosis. (3) BMD T-score below –2.5 (4) Follow-up ≥24 months. (5) Fracture at the thoracolumbar junction	(1) Neurological deficit associated with vertebral fracture. (2) Osteoporosis caused by other pathologic conditions. (3) History of spine surgery. (4) History of high-energy trauma	Preoperative + immediately postoperative + 12, 24 months postoperative	TK and SVA	Sagittal imbalance was a significant predictor for further collapse of a cemented vertebra and the progression of sagittal deformity in patients with a recollapsed vertebra following PVP for thoracolumbar OVF
Oishi et al. 2020 [25]	Presence or absence of adjacent vertebral fractures has no effect on long-term global alignment and quality of life in patients with osteoporotic vertebral fractures treated with balloon kyphoplasty	To investigate whether the presence or absence of AVF after PKP is correlated with the loss of global alignment and change in quality of life	Hamawaki Orthopaedic Hospital, Hiroshima, Japan	Longitudinal observational study	Medical records of consecutive OVF patients who underwent PKP 2012-2015. Age 80 (59-92) years	No AVF group: n=23 (19 W, 4 M)	T7-L3, only single fracture: no	KP	(1) Global sagittal alignment could be assessed preoperatively and throughout the follow-up. (2) Quality of life could be assessed preoperatively and throughout the follow-up	(1) Presence of tumour, infection, or any other disease that would induce pathological fractures. (2) History of lumbar fusion surgery. (3) Inability to maintain a standing position for whole-spine imaging. (4) Inability to attend follow-up due to comorbidities or institutionalization	Preoperative + 1 year postoperative + final follow-up (1-5 years)	SVA	The presence or absence of AVF did not affect long-term global alignment and patient quality of life. Loss of alignment after PKP was mainly due to AVF in the AVF (+) group, but due to recollapse of the BKP site in the AVF (- ) group, and both groups showed similar global alignment before PKP and at the follow-up
Pumberger et al. 2020 [26]	Kyphoplasty restores the global sagittal balance of the spine independently from pain reduction	To gain more detailed knowledge about the effect of PKP on the sagittal profile of the spine for a differentiated evaluation of the long-term risk/benefit ratio and to allow for more precise treatment recommendations for VCFs	Charité – Universitätsmedizin Berlin, Berlin, Germany	Longitudinal observational study	Consecutive patients with a single VCF, 2014- 2018. Age 70 ± 10.9 years	Overall: n=73 (47 W, 26 M)	Thoracic, lumbar, only single fracture: yes	KP	(1) Preoperative and postoperative upright whole spine radiographs <30 days before and after the operation. (2) Futile conservative treatment attempt	(1) Additional spinal operations. (2) New vertebral fractures between preoperative and postoperative radiographs. (3) Prior instrumented spinal fusion including L5/S1	Preoperative + 2.8 ± 3.3 days postoperative	TK, LL, SVA, SS, and PT	Our results show a significant improvement in the SVA in patients with vertebral fractures after KP. The change in SVA did not correlate with postoperative pain reduction. While guidelines mainly recommend considering KP only after a futile conservative treatment attempt, treating physicians should also keep in mind that patients (especially those with a sagittal imbalance) might benefit from a restored posture, irrespective of pain relief, and that this might only be possible for a specific time after fracture occurrence
Su et al. 2022 [27]	The short-term changes of the sagittal spinal alignments after acute vertebral compression fracture receiving vertebroplasty and their relationship with the change of Bathel Index in the elderly	To investigate the change of sagittal alignment using PVP and evaluate the outcomes with the BI	Hualien Tzu Chi Hospital, Hualien, Taiwan	Longitudinal observational study	Patients diagnosed with OVCF and treated in 2020. Age 80.7 ± 8.3 years. Three groups: the thoracic group (T1–T9), thoracolumbar group (T10–L2), and lumbar group (L3–L5), according to the location of the fractured vertebra. Two subgroups: AVF (+), AVF (-), no AVF	Overall: n=42 (34 W, 8 M)	T1-T9, T10-L2, and L3-L5, only single fractures: yes	Vertebroplasty	NA	(1) Previous spinal instrumentation surgery. (2) History of cancer. (3) Multiple fractures. (4) Cardiopulmonary disorders. (5) Spinal infection. (6) Pathologic fracture. (7) Difficulty standing straight	Preoperative + 2, 6 weeks postoperative + 3 months postoperative	VAS TK, LL, SVA, SSA, TPA, PI, PT, and SS	PVP for the elderly with acute VCFs in the subacute phase was effective for functional restoration and spine alignment improvement, and it was critical for quality-of-life recovery. In elderly patients with acute VCFs, PVP improved function and sagittal spinal alignment within 12 weeks, which was a crucial stage in their future recovery
Sutipornpalangkul et al. 2016 [28]	Comparison of sagittal balance between radiofrequency targeted vertebral augmentation and balloon kyphoplasty in treatment of vertebral compression fracture: A retrospective study	To compare the effectiveness of high-viscosity cement, RF-TVA, and PKP on spinal sagittal balance	Faculty of Medicine Siriraj Hospital, Bangkok, Thailand	Longitudinal observational study	Medical records of patients with single painful OVCF treated from 2007 to 2014. PKP group: Age 78.3 ± 6.1 years	PKP group: 17 (11 W, 6 M)	Levels not reported, only single fracture: yes	RF-TVA, KP	(1) Osteoporosis as the cause of VCF confirmed by biopsy. (2) Compression fracture vertebrae showed a high signal intensity on short T1 inversion recovery MRI and a low signal intensity on T1-weighted MRI	(1) Biopsy showed a pathological fracture. (2) Incomplete lateral whole spine radiography for radiographic measurement of spinopelvic parameters*	Preoperative + 1 week postoperative	LL, PI, SS, PT, SSA, and SVA	Treating OVCF with RF-TVA or PKP did not improve whole spine alignment
Yokoyama et al. 2015 [29]	Postoperative change in sagittal balance after Kyphoplasty for the treatment of osteoporotic vertebral compression fracture	To analyze the changes in total spinal alignment after PKP in VCF patients	Takeda General Hospital, Kyoto, Japan	Longitudinal observational study	Patients with osteoporotic VCFs were referred to a hospital because conservative treatment did not alleviate pain. Treatment period 2013-2014. Age 77.1 ± 5.8 years	Overall: n=21 (? W, ? M)	T11-L4, only single fracture: yes	KP	(1) VCF with 0–90% loss of vertebral body height on plain X-rays of the spine. (2) severe back pain associated with a single VCF refractory to analgesic medication administered for 2+ weeks. (3) VAS ≥5, interfering with the activities of daily living, or pain on tapping on the spinal process of the fractured vertebra. (4) Affected vertebral body showing a high signal intensity on STIR MRI and a low signal intensity on T1-weighted MRI	(1) Cannot maintain a standing position because of lumbar pain. (2) Impaired cardiopulmonary function. (3) Spinal metastatic cancer. (4) Evidence of radiculopathy. (5) History of spinal surgery	Preoperative + 1 month postoperative	LL, SVA, SSA, PI, PT, and SS	KP is helpful for the management of VCF patients, not only for relieving fracture-caused pain but also for improving sagittal balance through the alleviation of local kyphosis
Dong et al. 2009 [30]	Improvement in respiratory function after vertebroplasty and kyphoplasty	To study the changes in the respiratory function of patients with OVCFs after PVP and PKP	First Affiliated Hospital of Suzhou University, Suzhou, China	Non-randomized clinical trial	Patients with thoracic OVCFs treated in 2006-2008. Vertebroplasty: age 70.2 ± 5.5 years. KP: age 69.5 ± 6.4 years	Overall: n=38 (38 W, 0 M). Vertebroplasty: n=18. KP: n=20	T9-L3, only single fracture: no	Vertebroplasty, KP	(1) OVCFs	(1) Pulmonary diseases. (2) Infection of the respiratory passages within the last three weeks. (3) Previous surgery or fracture of the spine. (4) Significant scoliosis. (5) Neurological disorder. (6) Cardiac pacemaker	Preoperative + 3 days postoperative + 3 months postoperative	VAS VC, TLC, FVC, MVV, and TK	PVP and KP improve the lung function impaired by OVCFs, and KP is better at improving VC for thoracic OVCFs
Greven et al. 2017 [31]	Influence of radiofrequency kyphoplasty on pulmonary function	To investigate how using radiofrequency PKP for treating VCFs influences pulmonary function, reduces back pain and functional impairment, and achieves height restoration of the vertebral body	Universitätsklinikum Bonn, Bonn, Germany	Longitudinal observational study	Patients with one VCF each. Treatment period not mentioned. Age 70.4 ± 9.4 years. Three groups based on the location of the fracture: group 1: Th8-11, group 2: Th12-L2, and group 3: L3-L4	Overall: n=25 (14 W, 11 M). Group Th8-11: n=4 (? W, ? M), group Th12-L2: n=16 (? W, ? M), and group L3-4: n=5 (? W, ? M)	Thoracic, lumbar, only single fracture: yes	KP	NA	(1) High-energy trauma. (2) Known tumor involvement. (3) Osteonecrotic-, burst-, or pedicle fractures. (4) Previous surgical treatment for a VCF. (5) Rheumatic disorders or Paget’s disease. (6) Acute or chronic infections. (7) BMI >35. (8) Uncontrolled diabetes HbA1c >7%. (9) Severe cardiopulmonary disease. (10) Myelopathy. (11) Long-term steroid therapy	Preoperative + 1 month postoperative	VAS, ODI %FEV1, %REF	Radiofrequency KP increases vertebral body height, reducing pain and functional impairment. It also delivers a noticeable improvement in pulmonary function, mainly when the fracture is located in the central spinal region of the diaphragm
Lee et al. 2011 [32]	The effect of vertebroplasty on pulmonary function in patients with osteoporotic compression fractures of the thoracic spine	To determine the effect of PVP on pulmonary function in patients with OVCFs of the thoracic spine	Dongshin General Hospital, Seoul, Korea	Longitudinal observational study	Patients with intractable pain owing to osteoporotic compression fractures in the thoracic spine. Treatment period 2005-2006. Age 75.7 ± 7.6 years	Overall: n=72 (62 W, 10 M)	Thoracic, only single fracture: no	Vertebroplasty	(1) 65+ yrs. (2) Osteoporosis. (3) Intractable back pain of >2 weeks duration. (4) 1 or 2 acute vertebral fractures in the thoracic spine confirmed by MRI	(1) Neoplasm in the fractured vertebral body. (2) Pulmonary disease. (3) Previous cardiothoracic surgery. (4) Previous spine surgery. (5) Neurologic disorders. (6) Muscular disorders. (7) Thoracic scoliosis	Preoperative + 2 days postoperative + 1, 3 months postoperative	VAS %FEV1, %FVC, FEV1/FVC ratio TK	PVP improved pulmonary function immediately after the procedure in patients with OVCFs of the thoracic spine, and this improvement was maintained thereafter. Although the kyphotic deformity was not corrected, PVP effectively reduced back pain and improved pulmonary function, which was correlated with pain relief
Masala et al. 2014 [33]	Chronic obstructive pulmonary disease (COPD) patients with osteoporotic vertebral compression fractures (OVCFs): Improvement of pulmonary function after percutaneous vertebroplasty (VTP)	To investigate the changes in respiratory function in patients affected by COPD with single OVCFs treated with PVP	Fondazione Policlinico “Tor Vergata”, Rome, Italy	Longitudinal observational study	Patients with COPD affected by OVCFs.Treatment period 2011-2013. Age 71.4 (65-77) years	Overall: n=45 (16 W, 49 M)	Thoracic, only single fracture: yes	Vertebroplasty	(1) Single dorsal vertebral involvement. (2) Bone marrow oedema on MRI. (3) No intracanal bone fragments. (4) Refractory pain despite conventional medical treatment for 3+ months	(1) Radicular pain and symptoms suggestive of neurological involvement	Preoperative + 1 week postoperative + 3, 12 months postoperative	VAS %VC, %FVC, %FEV1	PVP improves restrictive ventilatory impairment in patients with moderate and severe COPD affected by single thoracic OVCFs. We recommend this treatment in the management of these patients
Sheng et al. 2015 [34]	Improvement in pulmonary function of chronic obstructive pulmonary disease (COPD) patients with osteoporotic vertebral compression fractures (OVCFs) after kyphoplasty under local anesthesia	To investigate respiratory function changes, spinal deformity, and pain scores in COPD patients with OVCFs after PKP. To determine if the changes in pulmonary function are related to the spinal deformity correction and pain scores	Soochow University-affiliated Ninth Hospital of Wuxi City, Wuxi City, China	Longitudinal observational study	Older patients affected by COPD with OVCFs in the thoracolumbar segment. Treatment period not reported. Age 71.2 ± 6.3 years	Overall: n=31 (? W, ? M)	T9-L2, only single fracture: no	KP	(1) Severe pain from the fracture. (2) Pain resistant to conservative treatment	(1) Radicular pain. (2) Neurologic disorders	Preoperative + 3 days postoperative + 3 months postoperative	VAS %FEV1, %FVC, %MVV, FEV1/FVC ratio	Although the most crucial goal of KP is to alleviate pain, this study clarified that KP also improves impaired lung function, which is directly related to the mortality and quality of life of COPD patients. There were no significant correlations between the PFT parameters, the LKA, the VAS scores, and the LKA
Tanigawa et al. 2008 [35]	Improvement in respiratory function by percutaneous vertebroplasty	To retrospectively investigate changes in respiratory function after PVP	Kansai Medical University Hirakata Hospital, Osaka, Japan	Longitudinal observational study	Patients who had VCFs caused by osteoporosis. Treatment period 2005. Age 72.0 (59-86) years. Subgroups by fracture location: Th group, Th + L group, and L group. Subgroups by number of treated vertebrae: single group and multiple group	Overall: n=41 (39 W, 2 M)	T6-L5, only single fracture: no	Vertebroplasty	(1) Back pain caused by VCF. (2) Pain on percussion of the vertebral spinous process	(1) Back pain attributed to myelopathy. (2) Radiculopathy resulting from stenosis of the vertebral canal or narrowing of the intervertebral foramen	Preoperative (+ 1 day postoperative) + 10 moths postoperative	%FVC, %FEV1	Although the most crucial goal of PVP is to alleviate pain, the present study clarified that PVP also improves decreased lung function. Such improvements could lower the incidence of pulmonary complications, thus increasing the clinical significance of PVP
Wu et al. 2018 [36]	Is balloon kyphoplasty a better treatment than percutaneous vertebroplasty for chronic obstructive pulmonary disease (COPD) patients with osteoporotic vertebral compression fractures (OVCFs)?	To test whether PVP and PKP can provide similar short-term improvements in respiratory function for COPD patients with single-segment OVCFs	China-Japan Friendship Hospital, Beijing, China	Non-randomized clinical trial	COPD patients with one OVCF. Treatment period 2013-2015. Vertebroplasty: age 73.6 ± 6.2 years. KP: age 71.5 ± 5.6 years	Overall: n=69 (51 W, 10 M). KP: n=31 (25 W, 6 M) and vertebroplasty: n=30 (26 W, 4 M)	T8-L4, only single fracture: yes	Vertebroplasty, KP	(1) Severe back pain from a fracture that was resistant to 2-week non-surgical management	(1) Pathologic fractures, fractures caused by high-energy traumas, previous spinal surgery or fracture, multiple-segment vertebral fractures. (2) Fractures involving neurological deficits. (3) Significant spinal deformity. (4) Severely disabled. (5) Respiratory tract infection within the last 3 weeks. 6) FEV1 <30% Pred FEV1. (7) Back pain due to myelopathy or radiculopathy	Preoperative + 1 week postoperative + 3, 12 months postoperative	VAS, ODI VC, FVC, %FEV1, and MVV	PVP and BKP both alleviate pain and accelerate mobility, which, in turn, improves pulmonary function. We observed a similar rate of cement leakage in both groups and a shorter operation time for PVP. Radiographically, BKP is favored in restoring the height of fractured vertebras and correcting kyphosis. These advantages of BKP in radiography are not correlated with improved spirometry parameters
Yang et al. 2007 [37]	Changes of pulmonary function for patients with osteoporotic vertebral compression fractures after kyphoplasty	To measure the pulmonary function, spinal deformity, and pain scores before and after treatment with PKP, and test for changes	The First Affiliated Hospital of Soochow University, Suzhou, China	Longitudinal observational study	Patients with OVCFs in the thoracolumbar segment. Treatment period not reported. Age 69.1 ± 5.2 years	Overall: n=30 (30 W, 0 M)	T9-L2, only single fracture: no	KP	(1) OVCF. (2) non-smoker	(1) Pulmonary diseases. (2) Previous surgery or fracture of the spine. (3) Significant scoliosis. (4) Neurologic disorder. (5) Cardiac pacemaker	Preoperative + 3 days postoperative, 1 month postoperative	VC, IC, RV, FRC, TLC, FVC, and MVV	Most lung function parameters (VC, IC, RV, and FRC) did not immediately improve with the KP procedure and ultimate spine realignment. After KP, sagittal realignment drastically and quickly improved

Appendix 3

Articles excluded during full-text review and reasons for exclusion.

**Table 10:** Studies excluded during full-text review and reason for exclusion

Study ID	Reference citation	Eligible participants?	Eligible intervention?	Eligible data?
Behrbalk 2016	Behrbalk E, Uri O, Folman Y, et al. Staged correction of severe thoracic kyphosis in patients with multilevel osteoporotic vertebral compression fractures. Glob Spine J. 2016;6:710-20. 10.1055/s-0035-1569460	Yes	No	Yes
Bo 2023	Bo J, Zhao X, Hua Z, et al.: Impact of sarcopenia and sagittal parameters on the residual back pain after percutaneous vertebroplasty in patients with osteoporotic vertebral compression fracture. Orthop Surg Res 2022;17:111. 10.1186/s13018-022-03009-4	Yes	Yes	No
Cavanilles-Walker 2022	Cavanilles-Walker JM, Rodriguez Montserrat D, Plano Jerez X, et al.: Sagittal imbalance influences outcome of vertebroplasty in patients with osteoporotic vertebral compression fracture. [Translated article] Rev Esp Cir Ortop Traumatol. 2022;66:T348-T354. 10.1016/j.recot.2021.04.002	Yes	Yes	No
Chen 2022	Chen Z, Yao Z, Wu C, et al.: Assessment of clinical, imaging, surgical risk factors for subsequent fracture following vertebral augmentation in osteoporotic patients. Skeletal Radiol. 2022;51:1623-30. 10.1007/s00256-022-04009-5	Yes	Yes	No
Cheng 2021	Cheng H, Wang GD, Li T, et al.: Radiographic and clinical outcomes of surgical treatment of Kümmell’s disease with thoracolumbar kyphosis: a minimal two-year follow-up. BMC Musculoskelet Disord. 2021;22:761. 10.1186/s12891-021-04640-8	Yes	No	Yes
Deen 2005	Deen HG, Aranda-Michel J, Reimer R, et al.: Preliminary results of balloon kyphoplasty for vertebral compression fractures in organ transplant recipients. Neurosurg Focus. 2005;18:e6. 10.3171/foc.2005.18.3.7	Yes	Yes	No
Griffoni 2020	Griffoni C, Lukassen JNM, Babbi L, et al.: Percutaneous vertebroplasty and balloon kyphoplasty in the treatment of osteoporotic vertebral fractures: a prospective randomized comparison. Eur Spine J. 2020;29:1614-20. 10.1007/s00586-020-06434-3	Yes	Yes	No
Iwata 2017	Iwata A, Kanayama M, Oha F, et al.: Does spinopelvic alignment affect the union status in thoracolumbar osteoporotic vertebral compression fracture? Eur J Orthop Surg Traumatol. 2017;27:87-92. 10.1007/s00590-016-1844-1	Yes	No	Yes
Kao 2019	Kao FC, Huang YJ, Chiu PY, et al.: Factors predicting the surgical risk of steoporotic vertebral compression fractures. J Clin Med. 2019;8:501. 10.3390/jcm8040501	Yes	Yes	No
Kim 2016	Kim YC, Bok DH, Chang HG, et al.: Increased sagittal vertical axis is associated with less effective control of acute pain following vertebroplasty. Bone Joint Res. 2016;5:544-51. 10.1302/2046-3758.511.BJR-2016-0135.R1	Yes	Yes	No
Korovessis 2007	Korovessis P, Repantis T, George P: Treatment of acute thoracolumbar burst fractures with kyphoplasty and short pedicle screw fixation: Transpedicular intracorporeal grafting with calcium phosphate: A prospective study. Indian J Orthop. 2007;41:354-61. 10.4103/0019-5413.37000	No	No	No
Korovessis 2008	Korovessis P, Zacharatos S, Repantis T, et al.: Evolution of bone mineral density after percutaneous kyphoplasty in fresh osteoporotic vertebral body fractures and adjacent vertebrae along with sagittal spine alignment. J Spinal Disord Tech. 2008;21:293-98. 10.1097/BSD.0b013e31812e6295	Yes	Yes	No
Kuo 2022	Kuo YR, Cheng TA, Chou PH, et al.: Healing of vertebral compression fractures in the elderly after percutaneous vertebroplasty—An analysis of new bone formation and sagittal alignment in a 3-year follow-up. J Clin Med. 2022;11:708. 10.3390/jcm11030708	Yes	Yes	No
Lee 2007a	Lee TO, Jo DJ, Kim SM: Outcome and efficacy of height gain and sagittal alignment after kyphoplasty of osteoporotic vertebral compression fractures. J Korean Neurosurg Soc. 2007;42:271-75. 10.3340/jkns.2007.42.4.271	Yes	Yes	No
Lee 2007b	Lee SB, Cho KS, Huh PW, et al.: Clinical and radiographic results of unilateral transpedicular balloon kyphoplasty for the treatment of osteoporotic vertebral compression fractures. Acta Neurochir Suppl. 2008;101:157-60. 10.1007/978-3-211-78205-7_27	Yes	Yes	No
Li 2014	Li D, Huang Y, Yang H, et al.: Jack vertebral dilator kyphoplasty for treatment of osteoporotic vertebral compression fractures. Eur J Orthop Surg Traumatol. 2014;24:15-21. 10.1007/s00590-012-1131-8	Yes	Yes	No
Liang 2020	Liang XJ, Zhong WY, Luo XJ, et al.: Risk factors of adjacent segmental fractures when percutaneous vertebroplasty is performed for the treatment of osteoporotic thoracolumbar fractures. Sci Rep. 2020;10:399. 10.1038/s41598-019-57355-1	Yes	Yes	No
Liu 2008	Liu JB, Tang XM, Xu NW, et al.: Preliminary results for the treatment of a pain-causing osteoporotic vertebral compression fracture with a sky bone expander. Korean J Radiol. 2008;9:420-25. 10.3348/kjr.2008.9.5.420	Yes	Yes	No
Liu 2022	Liu H, Zhou Q, Shao X, et al.: Percutaneous kyphoplasty in patients with severe osteoporotic vertebral compression fracture with and without Intravertebral cleft: A retrospective comparative study. Int J Gen Med. 2022;15:6199-6209. 10.2147/IJGM.S369840	Yes	Yes	No
Ma 2022	Ma CH, Yang HL, Huang YT, et al.: Effects of percutaneous vertebroplasty on respiratory parameters in patients with osteoporotic vertebral compression fractures. Ann Med. 2022;54:1320-27. 10.1080/07853890.2022.2063373	Yes	Yes	No
Matsumoto 2023	Matsumoto K, Hoshino M, Omori K, et al.: The relationship between global sagittal balance and the incidence of early adjacent vertebral fractures following balloon kyphoplasty. World Neurosurg. 2023;175:e818-22. 10.1016/j.wneu.2023.04.027	Yes	Yes	No
Papadopoulos 2008	Papadopoulos EC, Edobor-Osula F, Gardner MJ, et al.: Unipedicular balloon kyphoplasty for the treatment of osteoporotic vertebral compression fractures: Early results. J Spinal Disord Tech. 2008;21:589-96. 10.1097/BSD.0b013e31815d6997	No	Yes	No
Park 2017	Park JW, Park JH, Jeon HJ, et al.: Kümmell’s disease treated with percutaneous vertebroplasty: minimum 1 year follow-up. Korean J Neurotrauma. 2017;13:119-23. 10.13004/kjnt.2017.13.2.119	Yes	Yes	No
Park 2021	Park JS, Park YS: Survival analysis and risk factors of new vertebral fracture after vertebroplasty for osteoporotic vertebral compression fracture. Spine J. 2021;21:1355-61. 10.1016/j.spinee.2021.04.022	Yes	Yes	No
Pflugmacher 2005	Pflugmacher R, Kandziora F, Schroder R, et al.: [Vertebroplasty and kyphoplasty in osteoporotic fractures of vertebral bodies -- a prospective 1-year follow-up analysis]. Rofo. 2005;177:1670-76. 10.1055/s-2005-858631	Yes	Yes	No
Pflugmacher 2006	Pflugmacher R, Schroeder RJ, Klostermann CK: Incidence of adjacent vertebral fractures in patients treated with balloon kyphoplasty: Two years' prospective follow-up. Acta Radiol. 2006;47:830-40. 10.1080/02841850600854928	Yes	Yes	No
Phillips 2003	Phillips FM, Ho E, Campbell-Hupp M, et al.: Early radiographic and clinical results of balloon kyphoplasty for the treatment of osteoporotic vertebral compression fractures. Spine. 2003;28:2260-65; discussion 2265-67. 10.1097/01.BRS.0000085092.84097.7B	Yes	Yes	No
Plais 2023	Plais N, Bustos JG, Mahillo-Fernández I, et al.: Osteoporotic vertebral fractures localized in the lumbar area significantly impact sagittal alignment. Osteoporos Int. 2024;35:277-84. 10.1007/s00198-023-06936-y Note: Available online in December 2023.	Yes	No	No
Pradhan 2006	Pradhan BB, Bae HW, Kropf MA, et al.: Kyphoplasty reduction of osteoporotic vertebral compression fractures: Correction of local kyphosis Versus overall sagittal alignment. Spine. 2006;31:435-41. 10.1097/01.brs.0000200036.08679.1e	No	Yes	No
Röhrl 2006	Rohrl B, Sadick M, Brocker K, et al.: MDCT after balloon kyphoplasty: Analysis of vertebral body architecture one year after treatment of osteoporotic fractures. [German]. RoFo. 2006;178:801-9. 10.1055/s-2006-926872	No	Yes	No
Shibuya 2022	Shibuya Y, Katsumi K, Ohashi M, et al.: Effect of adjuvant therapy with teriparatide in patients with thoracolumbar osteoporotic vertebral fractures who underwent vertebroplasty with posterior spinal fusion. Sci Rep. 2022;12:8854. 10.1038/s41598-022-12655-x	Yes	No	Yes
Singh 2010	Singh K, Pflugmacher R: Directed cement flow kyphoplasty for treatment of osteoporotic vertebral compression fractures. In: Yue JJ, Guyer R, Johnson JP, et al. (eds). The Comprehensive Treatment of the Aging Spine: Minimally Invasive and Advanced Techniques. Philadelphia, PA: W. B. Saunders; 2010:243-47.	No	Yes	No
Spiegl 2019	Spiegl UJ, Anemuller C, Jarvers JS, et al.: Hybrid stabilization of unstable osteoporotic thoracolumbar vertebral body fractures: clinical and radiological outcome after a mean of 4 years. Eur Spine J. 2019;28:1130-37. 10.1007/s00586-019-05957-8	Yes	No	Yes
Sung 2023	Sung HS, Kim SI, Park HY, et al.: Predictive factors for conversion from conservatively to surgically treatment osteoporotic thoracolumbar compression fractures based on sagittal parameters and magnetic resonance imaging features. Eur Spine J. 2023;32:3933-40. 10.1007/s00586-023-07864-5	Yes	No	No
Takezawa 2012	Takezawa M, Takahashi T, Hanakita J, et al.: Early clinical and radiological results of balloon kyphoplasty in the treatment of ostoporotic vertebral compression fractures. Japanese Journal of Neurosurgery. 2012;21:959-66. 10.7887/jcns.21.959	Yes	Yes	No
Tanigawa 2012	Tanigawa N, Kariya S, Komemushi A, et al.: Added value of percutaneous vertebroplasty: Effects on respiratory function. AJR Am J Roentgenol. 2012;198:W51-54. 10.2214/AJR.11.6730	Yes	Yes	No
Topalidou 2015	Topalidou A, Tzagarakis G, Balalis K, et al.: Sagittal and frontal plane evaluation of the whole spine and clinical outcomes after vertebral fracturesAdv Orthop. 2015;2015:787904. 10.1155/2015/787904	No	No	No
Trieb 2020	Trieb K, Nittinger M: Timing of kyphoplasty influences the outcome: a prospective study. Sportverletz Sportschaden. 2020;34:42-7. 10.1055/s-0043-124989	Yes	Yes	No
Voggenreiter 2005	Voggenreiter G: Balloon kyphoplasty is effective in deformity correction of osteoporotic vertebral compression fractures. Spine. 2005;30:2806-12. 10.1097/01.brs.0000190885.85675.a0	Yes	Yes	No
Voggenreiter 2008	Voggenreiter G, Brocker K, Rohrl B, et al.: Results of balloon kyphoplasty in the treatment of osteoporotic vertebral compression fractures. Unfallchirurg. 2008;111:403-12. 10.1007/s00113-008-1453-5	Yes	Yes	No
Yang 2020	Yang JJ, Koo KH, Kim K, et al.: Efficacy of postural reduction of vertebral compression fracture with extension lateral radiograph before vertebroplasty. World Neurosurg. 2020;143:e430-41. 10.1016/j.wneu.2020.07.188	Yes	Yes	No
Yeh 2011	Yeh JH, Yang SC, Kao YH, et al.: Clinical and radiographic evaluation of balloon kyphoplasty using VCFX for osteoporotic vertebral compression fracture. Formosan J Musculoskelet Disord. 2011;2:94-8. 10.1016/j.fjmd.2011.06.003	Yes	Yes	No
Yi 2007	Yi WJ, Lee JH, Lee HG, et al.: Efficacy and safety of balloon kyphoplasty in the treatment of osteoporotic vertebral body compression fractures: Compared with vertebroplasty. J Korean Neurosurg Soc. 2007;42:112-17. URL: https://jkns.or.kr/journal/view.php?number=1680	Yes	Yes	No
Zhang 2017	Zhang YL, Shi LT, Tang PF, et al.: Correlation analysis of osteoporotic vertebral compression fractures and spinal sagittal imbalance. Orthopade. 2017;46:249-55. 10.1007/s00132-016-3359-1	Yes	No	Yes
Zhao 2022	Zhao C, Liu X, Wang Y, et al.: The effects of biomechanical factors on adjacent vertebral compression fractures after percutaneous kyphoplasty: a propensity score matching analysis. Osteoporos Int. 2022;33:1795-1806. 10.1007/s00198-022-06428-5	Yes	Yes	No
